# State of the art in osteoporosis risk assessment and treatment

**DOI:** 10.1007/s40618-019-01041-6

**Published:** 2019-04-12

**Authors:** J. Liu, E. M. Curtis, C. Cooper, N. C. Harvey

**Affiliations:** 1 MRC Lifecourse Epidemiology Unit, University of Southampton, Southampton General Hospital, Southampton, SO16 6YD UK; 2 NIHR Southampton Nutrition Biomedical Research Centre, University of Southampton and University Hospital Southampton NHS Foundation Trust, Southampton, UK; 3 NIHR Oxford Biomedical Research Centre, University of Oxford, Oxford, UK

**Keywords:** Osteoporosis, Epidemiology, Risk, FRAX, Fracture, Prevention

## Abstract

**Background:**

Osteoporosis constitutes a major public health problem, through its association with age-related fractures, particularly of the hip, vertebrae, distal forearm, and humerus. Over recent decades, it has evolved from being viewed as an inevitable consequence of ageing, to being recognised as a serious and eminently treatable disease.

**Materials and methods:**

In this article, we review the literature pertaining to the epidemiology of osteoporosis, associated health burden, approaches to risk assessment and treatment.

**Results:**

Although there is some evidence that fracture incidence has reached a plateau, or even started to decline, in the developed world, an ageing population and adoption of westernised lifestyles in transitioning populations is leading to an increasing burden of osteoporosis across the world. Whilst the clinical definition of osteoporosis has been based solely on bone mineral density, the prediction of fracture at the individual level has been improved by consideration of clinical risk factors in tools such as FRAX^®^, derived from a greater understanding of the epidemiology of osteoporosis. Such advances in approaches to primary and secondary prevention of fractures, coupled with elucidation of the underlying biology, and the development of a range of highly effective antiosteoporosis medications, have enabled a step change in our ability to prevent osteoporosis-related fractures. However, there remains a substantial disparity between the number of individuals at high fracture risk and number treated globally.

**Conclusion:**

Urgent work is needed at the level of health care systems, national and international policy, and in communication with patients and public, to ensure that all patients who should receive treatment for osteoporosis actually do so.

## Introduction

Osteoporosis is defined by the World Health Organization (WHO) as a ‘progressive systemic skeletal disease characterised by low bone mass and microarchitectural deterioration of bone tissue, with a consequent increase in bone fragility and susceptibility to fracture’ [1]. It is one of the major fundamental causes of fractures in individuals over the age of 50 years, with potentially serious and complex sequelae of comorbidities, both physical and psychological, and an associated increased relative mortality [1]. With the general secular trend towards an ageing population, there is an ever-increasing burden on time and healthcare costs spent on treating osteoporosis and associated fractures (termed ‘fragility fractures’) [1]. In this review, we report recent advances in the epidemiology, pathophysiology and treatment of osteoporosis, and approaches to risk assessment, highlighting current issues with regard to the globally apparent treatment gap.

## Epidemiology of osteoporosis

**Fig. 1:**
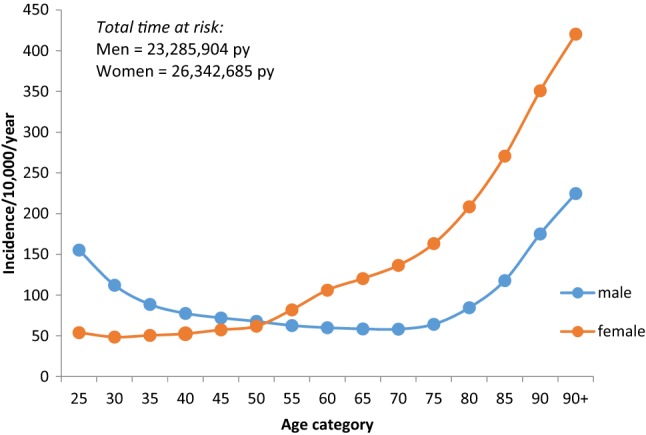
Incidence of any fracture by age and sex in the United Kingdom. Reproduced with permission from Curtis et al. [9]

The Global Burden of Disease study demonstrated a massive impact of musculoskeletal conditions on populations worldwide: the number of disability adjusted life years (DALYs) attributable to musculoskeletal disorders has increased by 17.7% between 2005 and 2013 [2], with osteoporotic fractures a major contributor [3]. The 2004 US Surgeon General’s report estimated that 10 million Americans over the age of 50 have osteoporosis, leading to 1.5 million fragility fractures each year [4], with another 34 million Americans at risk of the disease. Economically, the cost to the US is around $17.9 billion per annum. In the EU, a report estimated that, in 2010, 6.6% of men and 22.1% of women aged over 50 years had osteoporosis, and that there were 3.5 million fragility fractures [5]. The annual direct costs attributable to fracture treatment in the EU equate to approximately €24 billion, though, when indirect costs such as long-term care and facture prevention therapies are taken into account, this figure rises to €37 billion per year [5]. Osteoporotic fractures become more common with age, are more frequent in women than men at older ages (Fig. 1), and classically occur at sites such as the vertebrae, hip, wrist, humerus, scapula, ribs, and pelvis. In many Western populations, the risk of such a fracture occurring in the remaining lifetime from 50 years old is 50% for women and 20% for men [6].

### Variation in fracture rates across the world

**Fig. 2:**
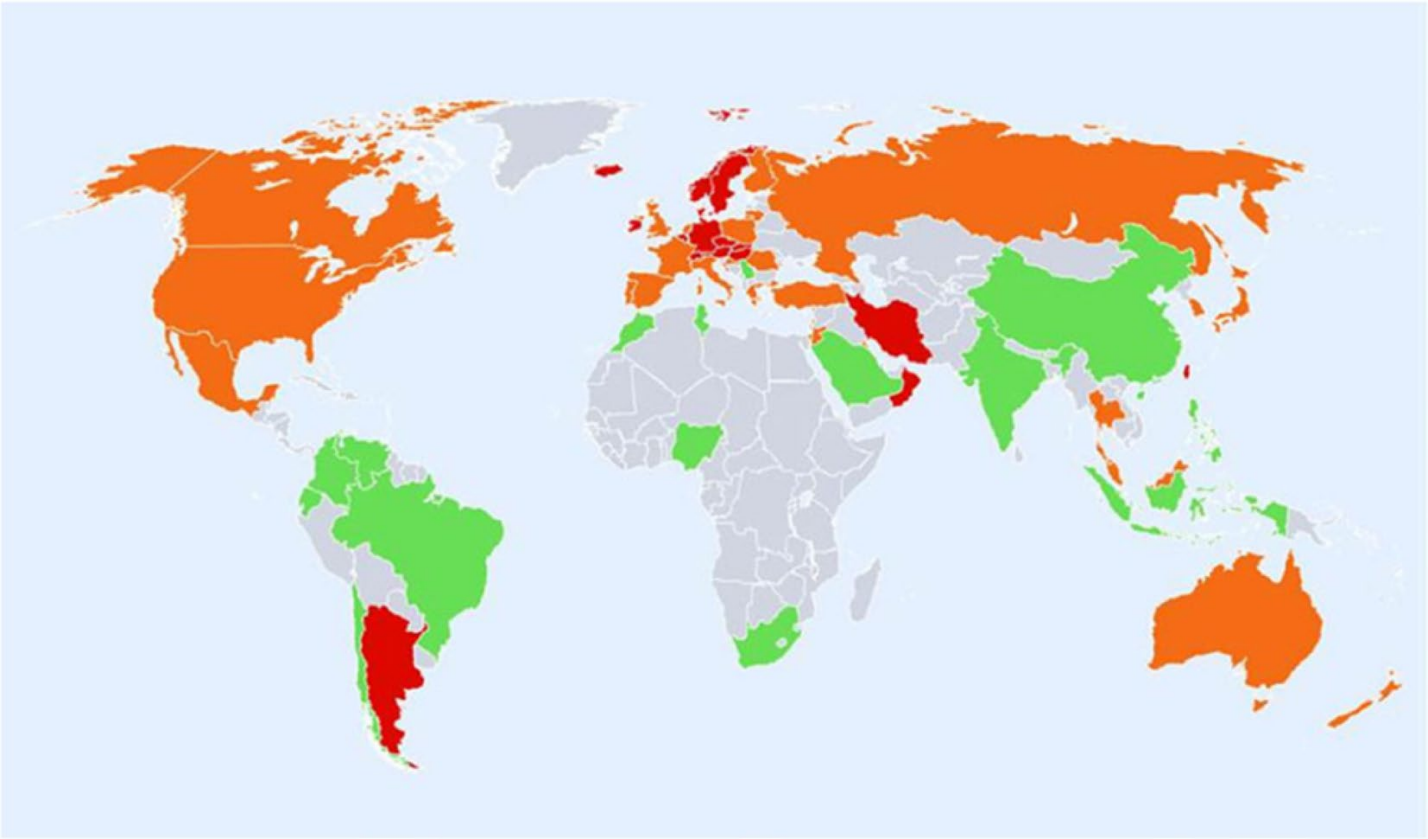
Hip fracture rates for men and women combined in different countries of the world categorised by risk; countries are coded red (annual incidence > 250/100,000), orange (150–250/100,000), or green (< 150/100,000) where estimates are available. Reproduced with permission from Kanis et al. [7]

Worldwide variation in fracture incidence is best documented for hip fracture, and studies have shown marked geographic differences in annual age-standardised hip fracture rates. The largest systematic review, published in 2012 [7], demonstrated that the highest annual age-standardised hip fracture incidences (per 100,000 person-years) were observed in Scandinavia [Denmark (574), Norway (563), and Sweden (539), plus Austria (501). The lowest were in Nigeria (2), South Africa (20), Tunisia (58), and Ecuador (73)]. In general, there was a series of high fracture risk countries in North Western Europe, Central Europe, the Russian Federation, and Middle-Eastern countries such as Iran, Kuwait, and Oman. Other high-risk countries were Hong Kong, Singapore, and Taiwan. In general, low-fracture-risk areas included Latin America (with the exception of Argentina), Africa, and Saudi Arabia, as shown in Fig. 2. Discounting the rates for Nigeria and South Africa, which were from either old or unreliable sources, there was around a tenfold range in hip fracture incidence worldwide; the overall age-standardised incidence in men was half that of women. In general, the highest incidence of hip fracture was documented in countries furthest from the equator and in countries in which extensive skin coverage due to religious or cultural practices is common, suggesting that vitamin D status may be an important underlying factor.

**Fig. 3:**
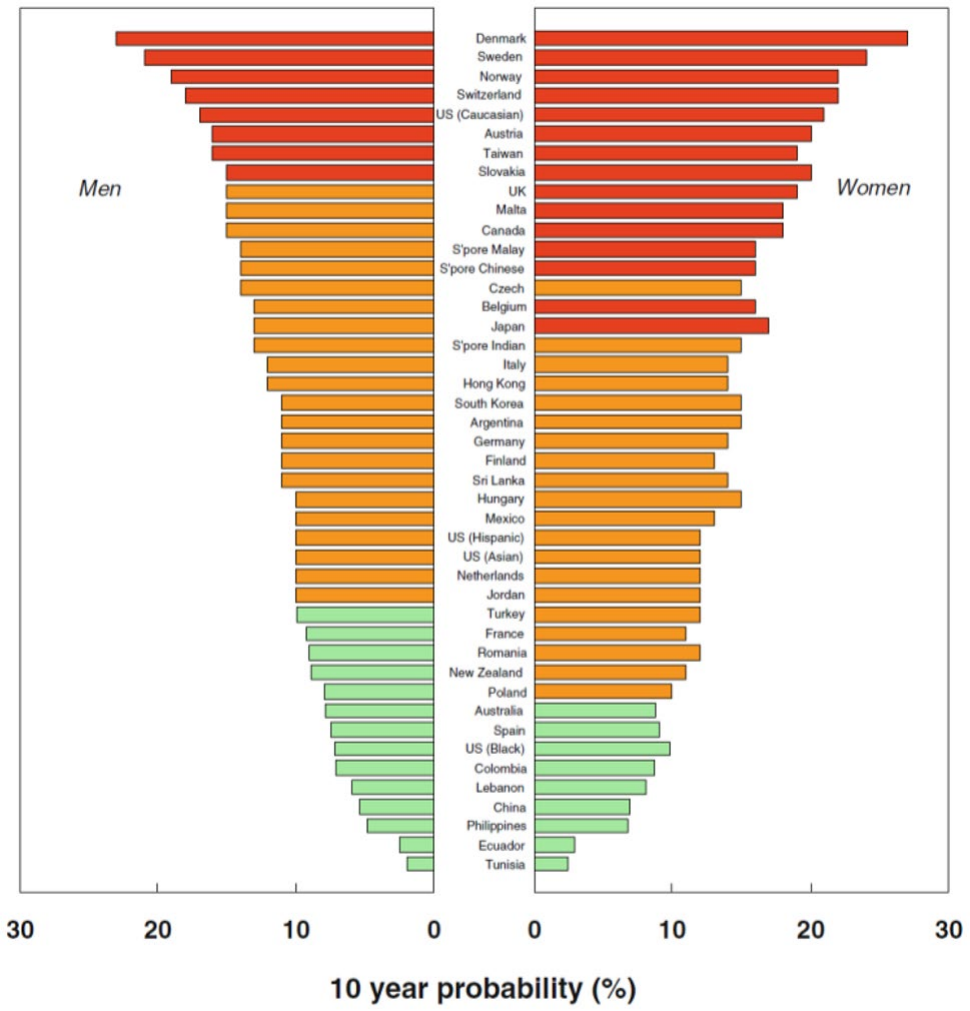
Ten-year probability of major fracture (in percent) in men and women aged 65 years with a prior fragility fracture and no other clinical risk factors, with a BMI of 24 kg/m^2^ at the threshold of osteoporosis as judged by BMD at the femoral neck (i.e., *T*-score − 2.5). Reproduced with permission from Kanis et al. [7]

The average 10 year probability of major osteoporotic fracture (hip, clinical vertebral, forearm, or humeral fracture) was calculated for those countries where an FRAX model was available. These fracture probabilities are shown in Fig. 3, demonstrating the marked variation in hip fracture risk by geographic location.

### Variation in fracture rates by ethnic group

**Fig. 4:**
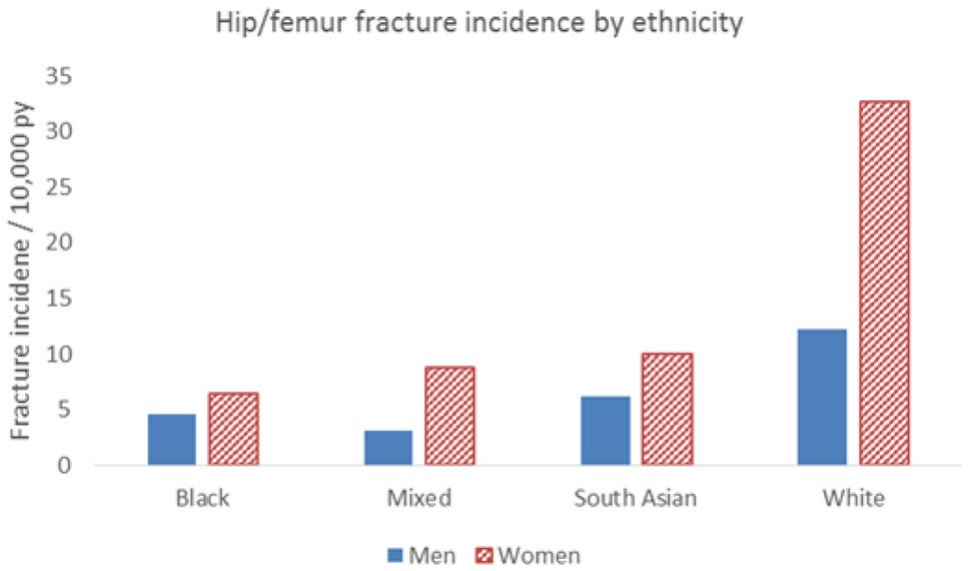
Incidence of hip/femur fractures by ethnicity in men and women aged over 50 years in the UK (Data from UK Clinical Practice Research Datalink, 1988–2012). Based on data from Curtis et al. [9]

As described previously, differences in fracture rates worldwide are partly attributable to ethnic differences in bone resistance to fracture. Studies in USA have demonstrated that the highest frequencies of hip fracture are observed in white women and the lowest in Black-American women [8]. Hip fracture rates in women of Hispanic and Asian ethnicity living in USA are lower than those observed in white women, but higher than Black women [8]. In a recent study conducted in the UK, the lowest rates of fracture were observed in black individuals; rates of fragility fracture in white women were 4.7 times greater than in black women and 2.7 times greater in white men than black men. Those of mixed or South Asian ethnicity had hip fracture rates of less than half that of individuals of white ethnicity (Fig. 4) [9]. This was consistent with studies comparing Dundee, Scotland and Johannesburg, South Africa [10], and within California, USA [11]. Lower BMD was noted in Chinese than Malay or Indian men [12] in Singapore, and lower BMD in Chinese and Malay women compared with Indian women [13]. Differences by ethnicity in skeletal size and microarchitecture, peak bone mineral density, and skeletal loss, in addition to differences in proximal femoral geometry are thought to underlie these variations in hip fracture rates [14, 15]. African–American women have higher areal BMD [16], greater bone area, increased trabecular thickness, cortical area and cortical thickness, and reduced cortical porosity compared to Caucasian women, all of which will confer greater bone strength and resistance to fracture, and persisted after adjustment for DXA BMD [14].

### Secular trends in fracture incidence

Current estimates suggest that 12% of the world population are over the age of 60 years—a total of around 901 million people. Europe has the greatest percentage of its population aged 60+ years (24%); however, rapid ageing in other parts of the world means that, by 2050, all continents except Africa will have 25% or more of their populations aged 60+ years. The number of older people in the world is projected to be 1.4 billion by 2030 and 2.1 billion by 2050, and could rise to 3.2 billion by 2100 [17].

This growth in the world population and the increasing proportions of older people will substantially impact the number of hip fractures globally in coming decades. A conservative estimate of the annual number of hip fractures suggests an increase from 1.66 million in 1990 to 6.26 million in 2050, with the latter figure potentially over 20 million when known secular trends are considered [18, 19]. Alterations in age- and sex-adjusted incidence rates have been documented most reliably for hip fracture [20]. Hip fracture rates appeared to have reached a plateau or even decreased in the last couple of decades in many developed countries, following a rise in earlier years; conversely in the developing world, age- and sex-specific rates are still rising in many areas [20]. A recent UK study demonstrated little change in fracture incidence overall from 1990 to 2012, though a small increase in male hip fracture rates was seen (10.8–13.4 per 10,000 person-years) [21]. In Asia, secular trends in hip fracture rates are variable: rates in Hong Kong appeared to have stabilised between 1985 and 1995, following an earlier steep increase [22]. Conversely, rates in Beijing have risen by around 33% between 1988 and 1992 from being among the lowest in the world, though this may be due to more accurate reporting in hospitals [23]. In Singapore, one of the most urbanised parts of Asia, hip fracture incidence increased by around 1% per year between 1991 and 1998 in comparison with rates derived from 1965 [24]. In Japan, ongoing age- and sex-specific increases in hip fracture rates of around 3.8% per year were recorded in 2006; a 32% increase in age- and sex-standardised fracture rates was observed between the periods 1992–1994 and 2010–2012 [25]. Such increases are consistent with observed rapid increases in urbanisation, associated with attendant changes in physical activity and nutrition.

Whilst the burden of osteoporosis can be assessed in terms of consequent fracture, there is value in identifying the number of individuals at high fracture risk to help to inform future health resource allocation. Using this approach, it has been estimated that, in 2010, there were 21 million men and 137 million women aged 50 years or greater at high fracture risk, and that this number is expected to double by 2040, with the increase predominantly borne by Asia [26]. Such increases in the burden of osteoporosis across the world highlight the need for effective primary and secondary prevention strategies, driven by fracture risk assessment.

## Identification of patients at high risk of fracture

It is apparent from the evidence described above, that osteoporotic fractures place a huge burden on societies across the world. Osteoporosis is a silent disease until a fracture occurs and patient perception of fracture risk is often underestimated [27, 28], so initiation of primary prevention is usually reliant on healthcare practitioners. It is unsurprising, therefore, that secondary prevention (identifying individuals for treatment on the basis of a low-trauma fragility fracture occurrence) is the paradigm most often used as the starting point for fracture prevention. However, whatever the approach to fracture risk reduction, it is critically important to place this within the context of local factors, such as the background fracture risk of the population, patterns of risk factors, funding constraints, and willingness of healthcare providers to pay for treatment.

### Secondary fracture prevention

Following attendance to a healthcare practitioner with a new fracture, it is important to assess fracture risk in a straightforward way, and to treat if appropriate. Several methods have been explored—some staff-based, some computer-based, and others a combination of the two. The most successful systems usually focus on a multi-disciplinary Fracture Liaison Service [29, 30], incorporating orthogeriatricians, rheumatologists, and fracture liaison clinical nurse specialists. The multi-disciplinary team, thus, ensures that medical management of patients admitted with fracture is optimised, both whilst in hospital, and for future fracture prevention, ideally with a lead clinician responsible for coordinating the group [31]. The International Osteoporosis Foundation has led the field internationally, with the institution of “a global campaign to facilitate the implementation of coordinated, multi-disciplinary models of care for secondary fracture prevention”. The “Capture the Fracture” (http://www.capturethefracture.org/) initiative has provided guidance on secondary fracture prevention, and also a global map, with a quality grading scheme, on which, subject to application, secondary fracture prevention services can be documented [32]. There is currently huge variation, not only between, but also within countries, and in the availability, scope, and quality of secondary prevention facilities. For example, a prospective observational study of over 60,000 older women recruited from primary care practices in ten countries showed that more than 80% of women with a fragility fracture did not receive osteoporosis treatment [33]. The Capture the Fracture initiative, aimed at raising the quality and coverage of fracture liaison services providing secondary prevention for osteoporosis, should provide a clinically valuable and cost-effective contribution to service improvement [34]. Importantly, the Fracture Liaison Service approach is associated with increased use of antiosteoporosis medications, reduced risk of subsequent fractures, and mortality [35, 36]. Further important initiatives around case finding of fragility fractures centre around vertebral fractures: around 12% of post-menopausal women with osteoporosis have at least one vertebral deformity, with less than a third of these individuals coming to clinical attention [37]. Primary care-based screening [38] and history-taking strategies distinguishing back pain likely to relate to vertebral fracture from other types of back pain may facilitate detection of these fractures [39]. In addition, consistent reporting of radiographs, CT scans, and the incorporation of vertebral fracture assessment in DXA scans will help with secondary fracture prevention in individuals with prevalent osteoporotic vertebral fracture.

### Primary fracture prevention

**Fig. 5:**
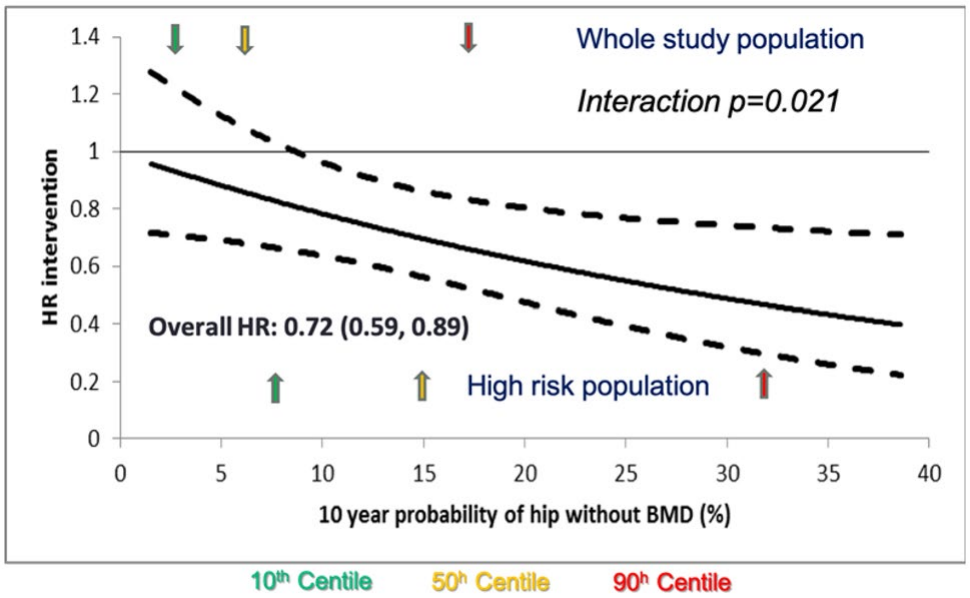
Systematic screening of older women for fracture risk in primary care leads to a reduction in hip fracture incidence (SCOOP study). Impact of screening on hip fracture compared with control arm, expressed as hazard ratio, across range of FRAX 10-year hip fracture probabilities at baseline, calculated without BMD. There was evidence of an interaction of effectiveness with baseline probability (*p* = 0.021). The symbols indicate the range of baseline probabilities in the whole-study population (closed symbols) and in the high-risk group identified by screening (open symbols). Modified from McCloskey et al. with permission [47]

In osteoporosis, as in any non-communicable chronic disease, there is clearly a balance between the benefits of a systematic screening approach leading to widespread treatment, with associated increased cost and risk of side effects, and a case-finding strategy focused on those at greatest individual risk, with associated problems of under-treatment. DXA screening is standard in the US (at the age of 65 years in women, and age 70 in men, and in individuals over the age of 50 years who have suffered an adult fracture) [40], but, in the majority of other countries, population screening is not judged to be cost-effective and primary prevention is focused more on opportunistic case finding, triggered by the presence of clinical risk factors [41–44]. A seven-centre randomised-controlled trial of the effectiveness and cost-effectiveness of screening older women in primary care for the prevention of fractures (the UK SCOOP study), in which approximately 12,500 older women were randomised to either normal care or screening and subsequent treatment (based on the FRAX risk assessment tool), has recently demonstrated that this intervention leads to a 28% reduction in hip fracture risk [45, 46]. As would be expected from the approach, screening appeared most effective in those at highest baseline fracture risk (since these were the individuals targeted for treatment, Fig. 5) [47], and importantly, was shown to be cost-effective [48].

## Tools for osteoporosis risk assessment

### Measurement of BMD alone

The WHO operational definition of osteoporosis is based on a DXA measurement of BMD, based on the clear link between lower BMD and increased fracture risk [49]. In recent years, however, it has been increasingly recognised that low BMD may be viewed as a risk factor for fragility fracture rather than as a disease in itself. Furthermore, other features independent of BMD, such as clinical risk factors, contribute substantially to fracture risk. A small proportion of the population is identified by a *T*-score of − 2.5 or below, and in terms of total numbers, more fractures in later life may occur in individuals who have a BMD in the normal or osteopenic than osteoporotic range. For example, in a study of 8065 post-menopausal women in USA, 243 women experienced a hip fracture over the 5 year study period, and only 46% of these women had a *T*-score ≤ − 2.5 at baseline screening [50]. Newer techniques such as peripheral quantitative computed tomography (pQCT) and HR-pQCT can provide a more detailed assessment of bone structure. However, their use in clinical practice is limited by the expense and availability of instruments, a lack of population-based reference data, and, indeed, any convincing evidence of their superiority, in terms of risk stratification, over traditional densitometry.

### Fracture risk assessment tools encompassing BMD and clinical risk factors

The use of clinical risk factors (CRFs) in addition to BMD measurement increases the accuracy of osteoporotic fracture risk assessment [51]. As such, a number of tools have been developed to calculate an individual’s risk of fracture, either based on clinical risk factors alone (QFracture) or in combination with BMD measurement (FRAX, Garvan). The most widely used tool is the Fracture Risk Assessment Tool, FRAX^®^ (http://www.shef.ac.uk/FRAX) [52], which has been developed across a large number of population-based cohorts worldwide. Two calculators developed from single cohorts are also available: The Australian Garvan Fracture Risk Calculator https://www.garvan.org.au/bone-fracture-risk and QFracture (http://www.qfracture.org) [53].

### Frax^®^

FRAX^®^ was developed by the then WHO Collaborating Centre for Metabolic Bone Diseases at the University of Sheffield, UK, and is the most comprehensively evaluated risk assessment tool currently available [52]. It integrates the risk of fracture with risk of death, to estimate the 10-year probability of major osteoporotic (clinical vertebra, hip, forearm, and proximal humerus) and hip fracture for individuals between the ages of 40–90 years. Clinical risk factors were selected on the basis of intuitive linkage to fracture risk, with at least partial independence from BMD, representing a risk that was amenable to pharmacological treatment and being readily available from standard clinical sources. BMD can be added into the tool if available. The tool was developed through a series of meta-analyses of prospective cohort studies from Europe, North America, Asia, and Australia including nearly 45,000 individuals, and has subsequently been validated in a similar number of individuals in other independent cohorts. Country (population)-specific FRAX calculators have since been developed to account for geographical variations in fracture incidence and mortality, incorporating inter-ethnic differences in risk within USA and Singapore, for example, taking into account migration effects [54]. The freely available Internet-based calculator is available in 34 languages; the fact that the model does not require BMD [55, 56] is of benefit to low resource settings where availability of DXA is limited. The website currently handles about 2.8 million calculations per year, but is not the sole portal for the calculation of fracture probabilities; for example, FRAX is available in BMD equipment, on smartphones and, in some countries, through handheld calculators (e.g., Poland and Russia) [54, 57]. In healthcare settings where trabecular bone score is available, this can also be incorporated into the fracture risk calculation.

Importantly, and self-evidently, not all CRFs for osteoporotic fracture are included in the FRAX tool (this being limited by which data were available globally in population-based cohorts) and many of the included CRFs have a dose–response element that is not incorporated into the model. Details of glucocorticoid exposure (e.g., dose and duration) were not available in the original FRAX cohorts, so that the relationship again assumes an average exposure; this will lead to an underestimation of fracture risk for recipients of higher daily doses of steroids and overestimation for low daily doses [58]. Based on the assumption that the average exposure in the FRAX cohorts probably lays within the range of 2–5–7.5 mg daily, an adjustment to the calculated fracture risk has been proposed based on the relative fracture risks according to steroid dose [59, 60]. Furthermore, although, as with all risk assessment tools, FRAX has not been validated in patients who have received antiosteoporosis treatment, there is evidence that it may still provide a useful guide in terms of continuation or cessation of therapy [61]. Further adjustment for differences between the femoral neck and lumbar spine BMD [62] and for past falls may also be made; indeed, whilst the lack of falls as an input variable has been a criticism of FRAX, the output probability has been shown to predict risk of incident falls [63]. Finally, the output FRAX probability can be modified to account for trabecular bone score [64].

### QFracture and Garvan fracture risk calculator

QFracture (UK) and the Garvan Fracture Risk Calculator (Australia) are risk assessment tools based on data from single countries, and generate a metric of cumulative fracture risk, as opposed to FRAX, which yields probability of fracture (considering the competing hazard of death). Thus, outputs are based on fundamentally different concepts. The Garvan calculator was derived using the Australian Dubbo cohort of around 2000 individuals, and includes men and women [65]. It yields absolute fracture risk as a percentage over 5 or 10 years for osteoporotic fracture or hip fracture, based on age, sex, prior fracture, falls, and bone mineral density. QFracture was developed using the apparently statistically driven identification of multiple clinical risk factors, many more than FRAX (30 in total and including falls), in a primary care database [53, 66]. Although the first version of QFracture was validated in an independent UK cohort [66], the second version (which now includes prior fracture) has been tested and validated in random subsets of the same overall cohort [53, 67], with further evidence of calibration in UK Clinical Practice Research Datalink (CPRD) [68]. It is critically important to realise that there are differences in the calibration of these instruments, particularly for major osteoporotic fracture, and thus, the outputs cannot be used interchangeably. Indeed, in the case of QFracture, there are several concerns with regard to calibration, its being based on a primary care data set, in which the prevalence of past fracture and family history of fracture are markedly lower than those expected from meta-analysis of comparable populations [69]. An example of a further specific concern is that, at the age of 85 years, the risk of hip fracture and major osteoporotic fracture (spine, humerus, distal forearm, and hip) are identical, the implication being that individuals of this age do not experience fractures of the spine, humerus, or distal forearm, a proposition that is somewhat at variance with clinical experience [69].

## Thresholds for intervention

Critically, none of the fracture risk assessment tools currently available directly yield an indication for treatment. Thus, the probability or risk generated needs to be interpreted, and thresholds set, above which pharmaceutical intervention is judged to be warranted. The cost-effectiveness of a therapeutic approach is often a key consideration in threshold setting.

There are two major approaches to the health economic assessment in a particular condition [70, 71]. First, one can assess the cost-effectiveness of the intervention, and set the threshold for intervention, for example FRAX probability, accordingly. Alternatively, one can derive a clinically informed and appropriate intervention threshold, and use cost-effectiveness analysis to validate a threshold. The 2017 National Institute for Health and Care Excellence (NICE) updated Multiple Technology Appraisal (MTA) on bisphosphonate use in osteoporosis [72] serves as an example of how, for a common disorder, the strict application of cost-effectiveness thresholds for relatively inexpensive drugs may lead to counter-intuitive and potentially harmful guidance [70, 73]. The widespread availability of low-cost generic forms of the main oral and intravenous bisphosphonates resulted in oral treatments being deemed cost-effective above a 1% risk of major osteoporotic fracture. Unfortunately, these were initially interpreted by some payers as clinical intervention thresholds, but, in fact, NICE directs practitioners to the UK National Osteoporosis Guideline Group (NOGG) guidance, which provides an illustration of the alternative approach to threshold setting. NOGG developed its guidance on the basis of clinical appropriateness, setting the threshold at the age-specific 10-year FRAX probability of fracture equivalent to women having already sustained a fracture. This approach, which avoids inappropriate over-treatment of older individuals and under-treatment of younger individuals, has been shown to be cost-effective [74], and has been adopted in many countries [75].

The approach to threshold setting varies substantially across the world, with guidelines using either fixed or variable age-dependent threshold, and, sometimes, combining a probability threshold with the requirement for BMD in the osteoporotic range [76]. Even between the USA and UK guidance, there is marked heterogeneity. The National Osteoporosis Foundation in USA suggests BMD assessment in women and men aged ≥ 65 years or 70 years, respectively, or at younger ages if they have had a prior fracture, and treatment for those with either a history of vertebral or hip fracture, osteoporosis on BMD assessment, or osteopenia and a 10-year FRAX-calculated probability of a hip fracture ≥ 3% or major osteoporotic fracture ≥ 20% [77]. Conversely, as mentioned above, the UK National Osteoporosis Guideline Group (NOGG) recommends the use of FRAX with or without BMD as the first step in risk assessment, with prior fragility fractures at older ages usually a sufficient basis for treatment regardless of other risk factors. Where a 10-year probability has been generated by FRAX, threshold graphs are subsequently used to guide appropriate intervention. The possible outcomes include patient reassurance with further risk calculation at a later date (low risk), BMD assessment (intermediate risk), or immediate treatment without the need for BMD assessment (high risk) [78]. Once BMD has been performed, the 10-year probability of fracture is plotted by age, either above or below a single treatment threshold, which is set at the 10-year fracture probability conferred by having had a previous fragility fracture, corresponding to older UK national guidance. The treatment threshold, thus, increases with age, but even so, the proportion of women potentially eligible for treatment rises from 20 to 40% across the age range assessed. A key message is that it should not be assumed that one size will fit all countries. For example, intervention in China at a threshold of 20% for FRAX major osteoporotic fracture, a threshold used in USA, would lead to only a very tiny proportion of the population treated [76]. Accordingly, the International Osteoporosis Foundation has published guidance relating to osteoporosis and corticosteroid-induced osteoporosis, which can be readily modified to reflect national priorities and subsequent treatment thresholds [41–43, 79].

## The osteoporosis treatment gap

**Fig. 6:**
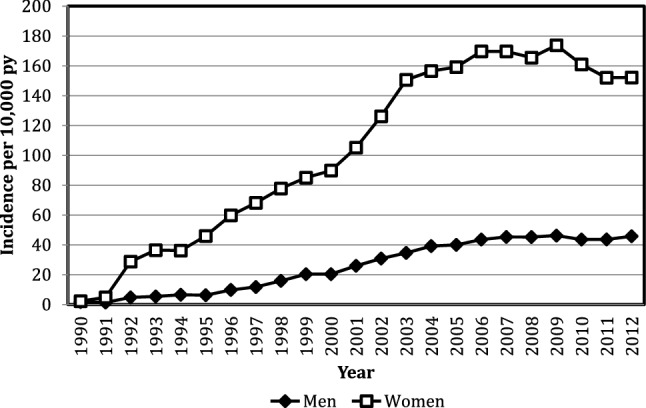
Incidence of antiosteoporosis medication prescription from 1990 to 2012 in the UK population aged 50 years or over. Reproduced with permission from van der Velde et al. [84]

Despite many advances in the diagnosis of osteoporosis, the assessment of fracture risk, the development of therapies to reduce the risk of fractures, and the production of best practice guidelines, many studies indicate that a minority of men and women at high fracture risk actually receive treatment. Even in patients who sustain a fragility fracture, fewer than 20% actually receive therapies to reduce the risk of fracture in the year following the fracture [80, 81], with particularly poor rates of treatment for older women and those who live in long-term care. Disparities in use of fracture risk assessment tools such as FRAX vary 1000-fold worldwide, with a far greater variability than the 30-fold range of crude, or tenfold range of age-standardised hip fracture worldwide, indicating a large gap in service provision. Limitations in access to the Internet, lack of national assessment guidelines for osteoporosis in many countries, and the availability of alternative assessment algorithms may partially explain these differences [54]. Not only is lack of assessment and lack of treatment of those at very high risk of further fracture a concern, most worrying is the downward trend in people being treated after hip fracture, demonstrated both in the USA and UK populations [82, 83]. A similar decline has been noted in the use of antiosteoporosis medications for primary prevention (Fig. 6) [84]. The precise causes for this trend are likely to be several, including the recent reimbursement changes in the US, and the massive inflation of concerns regarding potential rare side effects of long-term bisphosphonate treatment such as osteonecrosis of the jaw and atypical femoral shaft fractures (see below). Despite these events not even being definitively causally related to bisphosphonate treatment, and in absolute terms being very rare [85], media stories focusing on these outcomes have been common in recent years [86].

## Treatment of osteoporosis

### Vitamin D and calcium supplementation

A reasonably consistent interpretation of the often conflicting literature in this area is that calcium and vitamin D supplementation should generally be used together [87], and directed at those likely to be deficient in these nutrients or as adjunctive therapy with antiosteoporosis medications [88]. There is little evidence to support routine population-based supplementation, and overall, the potential effects of calcium and vitamin D on fracture reduction are modest and should not be viewed as a substitute for antiosteoporosis medications. Although one research group has suggested that calcium and vitamin D supplementation may be associated with increased cardiovascular risk [89], the evidence remains contradictory, and without established biological underpinnings [70].

### Bisphosphonates

Bisphosphonates are synthetic analogues of the naturally occurring compound pyrophosphate and bind strongly to hydroxyapatite, inhibiting bone resorption by inactivating osteoclasts. The most commonly prescribed oral bisphosphonate is oral alendronic acid. If taken correctly (in the morning with a glass of water, 45 min before food, drink, or other medications and remaining upright for about 30–60 min after the dose), upper gastrointestinal side effects are uncommon. However, for those who are unable to tolerate oral bisphosphonates, or in whom they are contraindicated (for example, malabsorption or dysphagia), then a buffered effervescent preparation of alendronic acid or and an intravenous bisphosphonate, such as zoledronic acid, are potential alternatives [90]. Although zoledronic acid has generally been used annually, a recent trial has demonstrated efficacy with a single infusion every 18 months in patients who have osteopenia rather than osteoporosis [91].

### Selective oestrogen receptor modulators (SERMs): raloxifene

Raloxifene is a selective oestrogen receptor modulator that has antiresorptive estrogenic effects on the skeleton without the unwanted effects of oestrogen in breast tissue. Indeed, raloxifene has been associated with a significant decrease in the risk of breast cancer. It is effective in preventing post-menopausal bone loss and reducing the risk of vertebral fractures. However, there is no evidence that raloxifene prevents hip or non-vertebral fractures [92]. Adverse effects include leg oedema, cramps, hot flushes, and a two-to-threefold increase in the risk of venous thromboembolism.

### Denosumab

Denosumab is a fully humanised antibody to receptor activator of nuclear factor kappa B ligand (RANKL) is a newer antiresorptive agent. RANKL, secreted by osteoblasts, is a major activator of osteoclastic bone resorption and mimics the action of osteoprotegerin (OPG) [90, 93]. It is administered as a subcutaneous injection once every 6 months and its efficacy has been demonstrated in patients with renal disease, although underlying renal bone disease should be considered in severe renal impairment. Administration of denosumab leads to increased BMD and reduction in risk of vertebral, non-vertebral, and hip fractures [94]. Side effects are uncommon, but may include skin infections, predominantly cellulitis. This is not typically seen at the injection site and is thought to be secondary to an immunomodulatory effect of the drug. Hypocalcaemia can also be a risk where there is concomitant renal impairment, particularly if the patient is vitamin D deficient.

### Teriparatide

Teriparatide (recombinant human 1–34 parathyroid hormone peptide) was the first truly anabolic (bone forming) agent. It is administered by subcutaneous injection in daily doses of 20 μg. It increases bone formation and produces large increases in BMD, leading to approximately 70% reduction in the incidence of new moderate or severe vertebral fractures over 18 months of treatment, together with reductions in non-vertebral fractures, compared with placebo [95–97]. Superiority in terms of BMD gain and fracture reduction has been recently demonstrated in comparison with oral risedronate [98]. Side effects are uncommon, but may include nausea, headache, and dizziness; in addition, transient hypercalcaemia and hypercalciuria may occur. There may be synergistic benefits through the use of combination treatments such as teriparatide plus denosumab or teriparatide plus zoledronic acid compared with use of these agents alone [99], although such approaches are not yet widely used or approved.

### Abaloparatide

Abaloparatide is a synthetic 34 amino acid peptide that shares structural homology with parathyroid hormone-related peptide [100]. It activates the same PTH-1 receptor as does teriparatide but with a greater affinity for the RG receptor configuration. Use of abaloparatide results in substantial gains in BMD and a reduction in both the vertebral (86% relative reduction) and non-vertebral fractures (43% relative reduction) after 18 months. Indeed, the effect on major osteoporotic fracture risk reduction appeared greater with abaloparatide than teriparatide. As with teriparatide, there is an increased likelihood of hypercalcaemia compared with placebo, and in the US (abaloparatide is not available in Europe), both are currently used in a similar clinical setting.

### Romosozumab

Sclerostin, an osteocyte-derived glycoprotein that modulates bone formation by osteoblasts, is primarily regulated by mechanical loading; increased load reduces sclerostin secretion [101]. By binding to LRP5/6, sclerostin inhibits the activation of the canonical Wnt signaling pathway, thus, inhibiting bone formation. Romosozumab is a humanised antibody that binds sclerostin with high affinity, and leads to dramatic increases in bone density. In a phase 3 fracture endpoint trial that enrolled 7180 women with post-menopausal osteoporosis, romosozumab 210 mg monthly for 12 months reduced the incidence of vertebral fracture by 73% [101]. This effect was particularly evident during months 7–12 of therapy. During the second year of the study, all patients received open label denosumab therapy. At the end of that year, vertebral fracture risk was reduced by 75% in patients who had received romosozumab during year 1 compared to the group that received placebo followed by denosumab. Clinical fracture risk was reduced by 36% compared to placebo after 12 months. The incidence of non-vertebral fracture was reduced by 25%, but this decrease was not statistically significant. Efficacy was further demonstrated in a trial of higher fracture risk patients, against alendronic acid as the comparator [102]. However, here, there was a modest imbalance in cardiovascular events (greater with romosozumab). At the time of writing, romosozumab is under consideration by the US FDA and the European Medicines Agency, and has just been licensed in Japan.

### Adverse effects and duration of therapy

Atypical femoral factures of the subtrochanteric region and femoral shaft may rarely occur in patients taking bisphosphonates or denosumab. These are usually located in the lateral cortex around which endosteal thickening may be observed prior to fracture occurrence. Individuals may have prodromal pain and fractures typically are transverse, sometimes bilateral, and occur after minimal trauma. Although these fractures can occur in bisphosphonate/denosumab naïve individuals, they appear more commonly in patients taking these therapies for a prolonged duration. It is thought that the reason for this increased incidence is related to over-suppression of bone turnover. Overall, the fractures prevented greatly outnumber those atypical events potentially resulting from medication [103]. Reassuringly, a recent study in the Danish population has demonstrated that users of alendronate still have a reduced risk of fracture compared with matched controls even after 10 years use, and that the number of hip fractures prevented is still greater than the number of subtrochanteric fractures occurring even by the end of a decade of bisphosphonate treatment [104]. Osteonecrosis of jaw is extremely rarely observed during therapy for osteoporosis (< 1/100,000/year) for individuals on oral bisphosphonates [105]), but appears more commonly when higher doses of bisphosphonates are given intravenously for treatment of bone metastases. A causal link to bisphosphonates is unproven, but international guidance suggests a prudent approach, encouraging patients to maintain good oral hygiene and have regular dental visits, with invasive dental work performed before commencement of bisphosphonate or denosumab therapy [106].

Current UK guidance has, therefore, moved towards a reassessment of the need for treatment after 3 years of intravenous bisphosphonate/subcutaneous denosumab, and 5 years of oral bisphosphonate [78]. For high-risk patients (examples might include those with a history of several fragility fractures, very low BMD, high FRAX probability, or using high-dose corticosteroids), continuation of treatment is usually warranted, but, in other situations, for example where there have been no incident fractures and bone mineral density has improved, a period without treatment may be considered, prior to further risk assessment and potential subsequent recommencement of treatment.

## Conclusion

Fractures associated with osteoporosis are common, and the number of individuals at high fracture risk is set to double globally over the next 3 decades. Effective approaches to primary and secondary fracture prevention have been established, and we have a range of effective pharmacological agents which improve bone mineral density and reduce the risk of incident fractures. However, there is still a substantial disparity worldwide between the number of patients who should be treated for osteoporosis and the number who actually are. Major international efforts are ongoing under the auspices of organizations such as the International Osteoporosis Foundation, but efforts are needed at all levels nationally and internationally to ensure that all patients at high fracture risk are assessed and treated appropriately.
